# Insights from the molecular docking analysis of GRP78 with natural compound inhibitors in the management of cancers

**DOI:** 10.6026/97320630019039

**Published:** 2023-01-31

**Authors:** Aisha Elaimi, M Baeissa Hanadi, Abdulrahman Almutairi, Rashed Ahmed Alniwaider, Munawir Alanazi Abulkaliq, Ahmed Shaker Naga, Juma Alkhenaizi Kadhem, Alam Qamre

**Affiliations:** 1 Department of Medical Laboratory Technology, College of Applied Medical Science, King Abdulaziz University, Jeddah, Saudi Arabia; 2 Centre of Innovation in Personalized Medicine, King Abdulaziz University, Jeddah, Saudi Arabia; 3 Department of Biochemistry, College of Science, University of Jeddah, Jeddah, Saudi Arabia; 4 Department of Pathology and Laboratory Medicine, King Abdulaziz Medical City, Ministry of National Guard Health Affairs (MNGHA), P.O. Box 22490, Riyadh, Saudi Arabia; 5 Department of Respiratory Services, Ministry of National Guard Health Affairs (MNGHA), P.O. Box 22490, Riyadh, Saudi Arabia; 6 Clinical Pathology Department, ExpressMed Laboratories, Block, 359, Zinj, Kingdom of Bahrain; 7 Molecular Genomics and Precision Medicine Department, ExpressMed laboratories, Block, 359, Zinj, Kingdom of Bahrain

**Keywords:** Cancer, GRP78, invasion, metastasis, natural compounds

## Abstract

Cancer is regarded as one of the world's most serious health issues. Glucose regulated protein (GRP78) exhibits a vital role in the proliferation, invasion, and metastasis of numerous cancer cells. Based on that, this study screened the 390 natural compounds targeting the GRP78 catalytic site. Among them, corynanthin, toyocamycin, and nanaomycin were found to strongly bind with GRP78 and possess the binding affinities of -8.4, -8.9, and -8.7 kcal/mol, respectively. In addition, these compounds interacted with key residues of GRP78 and have several amino acid residues interaction in common with the cocrystal ligand (ATP). Based on physicochemical parameters and ADME evaluations, these compounds were found to have good drug-like properties. These compounds could be used as possible GRP78 inhibitors in the fight against cancers. Albeit, exhaustive experimental studies would be required to confirm the findings described here.

## Background:

Cancer is regarded as one of the world's most serious health issues [1,2]. Cancer, in its most basic form, is defined as the uncontrolled division of aberrant cells. GRP78 exhibits a vital role in the proliferation, invasion, and metastasis of numerous cancer cells, including hepatoma cells [3], gastric cells [4], endometrial cells [5], lung cancer [6], prostate cancer [7], and breast cancer [8]. Glucose regulated protein (GRP78) is a mature endoplasmic reticulum (ER)-resident chaperone that belongs to the vast chaperone family of heat-shock protein 70 molecules [9]. Cancer cells have multiple molecular chaperones on their surface, including GRP78, which is normally found in the ER. Because this display is unique to cancer cells, these chaperones are important targets for therapeutic development. GRP78 overexpression can stimulate the development of MMPs (matrix metalloproteinases), as well as pancreatic cancer metastasis and invasion, via activating the c-Jun N-terminal kinase and focal adhesion kinase pathways [10]. However, GRP78 deletion not only decreased MMP expression but also hindered the RhoA signaling pathway, preventing tumor invasion [11]. CRIPTO or GRP78 knockout can inhibit cancer cell invasion, hence lowering cell proliferation, migration, colony formation, and other activities [7]. All these studies showed that GRP78 is a therapeutic target in the management of cancer. Computer-assisted drug design (CADD) has emerged as a powerful tool for discovering prospective lead compounds and assisting in the development of new medications for a wide variety of ailments [12]. CADD can help researchers investigate compound-receptor interactions. A variety of CADD techniques are now being utilized to find possible lead compounds from massive compound libraries [13]. The aim of this work was to uncover new promising leads from the natural compounds database utilizing in silico methodologies that might be employed as GRP78 inhibitors to fight cancers.

##  Methodology:

## Protein preparation:

The crystal structure of GRP78 ATPase domain in complex with ATP was obtained from PDB (PDB ID: 5F1X). The co-crystal ligand was removed and the protein was saved in .pdb format.

## Compounds library preparation and virtual screening:

We selected a library of natural products compounds consisting of 390 compounds retrieved from The national cancer institute's (NCI) development therapeutics program (DTP), which offers resources and assistance to research communities around the world to accommodate the exploration and the creation of novel cancer therapeutics. All the compounds were minimized and prepared using Discovery Studio 2021. AutoDock Vina 1.1.2 [14] and AutoDock 4.2.5.1 [15] were used for virtual screening and in-depth molecular docking analysis. X, Y, and Z values were set as 17.63, -5.61, and 4.94, respectively.

## Physiochemical and ADME properties:

Lipinski's rule was employed to filter the compound library, expelling compounds that did not meet the specified criteria; it is a method for assessing chemical compound drug-likeness and oral bioactivity. The regulations are designed to address ADME concerns [16]. The DataWarrior tool was utilized in order to make predictions regarding the safety and efficacy profiles of the top compounds that were screened [17].

## Results and Discussion:

**Figure 1:**
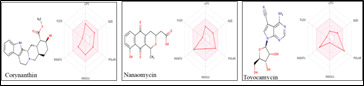
2D structure and bioavailability radar of top 3 compounds.

In this study, 390 natural compounds were screened against the active site of the GRP78. These compounds have already been listed as anticancer compounds in the NCI database. Thus, this study follows a drug repurposing approach to identify the new potential inhibitor targeting GRP78. The physicochemical and drug-likeness of 11 selected compounds were predicted, demonstrating their potential as lead molecules. All seven compounds were found to be the most acceptable because they exhibited no mutagenic, tumorigenic, reproductively effective, or irritant properties, as well as a significant drug score and drug-likeness (Table 2). Based on binding affinity (BA) values top 3 compounds (corynanthin, toyocamycin, and nanaomycin) were selected for in-depth studies. 2D structure and bioavailability radar of the top 3 compounds is demonstrated in Figure 1 for a rapid appraisal of drug-likeness. Lipophilicity, size, polarity, solubility, flexibility, and saturation are the six physicochemical properties of the bioavailability radar [18]. These predictions demonstrated that all these compounds have the optimum values and are within the range, indicating that they are potential lead molecules.

**Figure 2:**
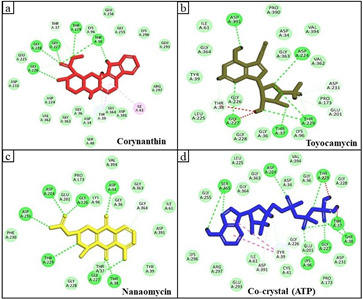
Interacting amino acid residues of a) corynanthin, b) toyocamycin, c) nanaomycin, and d) ATP with GRP78.

**Figure 3:**
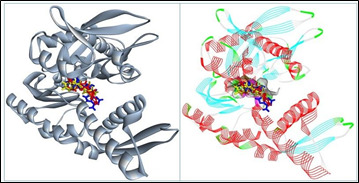
Superimposition view of corynanthin, toyocamycin, nanaomycin, and ATP in the catalytic site of GRP78. Corynanthin, toyocamycin, nanaomycin, and ATP are shown in red, dark yellow, yellow, and green color, respectively.

**Figure 4:**
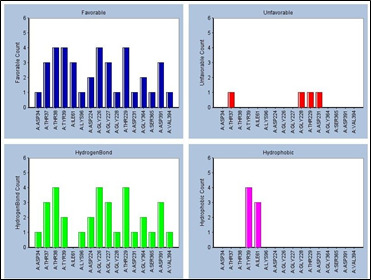
Residue interaction histograms

Corynanthin interacted with Asp231, Gly226, Leu225, Gly228, Gly227, Thr37, Thr229, Lys96, Thr38, Gly255, Glu256, Lys296, Glu293, Arg297, Ile61, Asp391, Gly364, Tyr39, Asp34, Gly36, Gly363, Asp224, and Val362 residues of GRP78. Gly226, Gly228, Gly227 and Thr38 residues of GRP78 H-bonded with corynanthin (Figure 2a). Toyocamycin interacted with Asp391, Pro390, Asp34, Val394, Gly363, Asp224, Val362, Asp231, Pro173, Glu201, Thr229, Lys96, Thr37, Gly36, Gly228, Gly227, Gly226, Thr38, Leu225, Tyr39, Gly364, and Ile61 residues of GRP78. Asp391, Asp224, Thr229, Thr37 and Gly227 residues of GRP78 H-bonded with toyocamycin (Figure 2b). Nanaomycin interacted with Asp231, Asp224, Glu201, Gly226, Pro173, Lys96, Asp34, Val394, Gly36, Gly363, Gly364, Ile61, Asp391, Tyr39, Thr38, Thr37, Gly227, Gly228, Thr229, and Phe230 residues of GRP78. Asp231, Asp224, Gly226, Asp34, Gly227, Thr38, Gly227, and Thr229 residues of GRP78 H-bonded with nanaomycin (Figure 2c). Thr37, Thr38, Glu293, Lys296, Ser300, Arg367 have been shown as the key ATP binding site interacting residues [19]. Interestingly, corynanthin, toyocamycin, and nanaomycin have been found to interact with these residues. BAs of corynanthin-GRP78, toyocamycin-GRP78, and nanaomycin-GRP78 complexes were found to be -8.4, -8.9, and -8.7 kcal/mol, respectively (Table 1). The cocrystal ligand (ATP) interacted with Ser365, Gly364, Gly363, Leu225, Asp224, Asp34, Gly36, Val394, Thr229, Gly228, Thr37, Thr38, Gly227, Asp231, Glu201, Lys96, Pro173, Tyr39, Cys41, Asp391, Ile61, Glu293, Arg297, Lys296, and Gly225 residues of GRP78 (Figure 2d). Interestingly, several amino acid residues of GRP78 were common in interaction with the hit compounds (corynanthin, toyocamycin, and nanaomycin) and the ATP. In addition, the superimposition view showed that the binding patterns of corynanthin, toyocamycin, and nanaomycin in the GRP78 active site were similar to those of the ATP (Figure 3)(Figure 4).

## Conclusion:

Corynanthin, toyocamycin, and nanaomycin were found to tightly bind with GRP78, interacted with key residues of GRP78, and have several amino acid residues interaction in common with the cocrystal ligand (ATP). These compounds could be used as possible GRP78 inhibitors in the fight against cancers. Albeit, exhaustive experimental studies would be required to confirm the findings described here.

## Figures and Tables

**Table 1:** List of top-screened compounds

Serial No.	Compound name	Binding affinity (kcal/mol)
1	Toyocamycin	-8.9
2	Nanaomycin	-8.7
3	Corynanthin	-8.4
4	Ehnahydrochloride	-8.4
5	Medicarpin	-8.3
6	Pentoxifyllin	-8.2
7	Taxifolin	-8.1
8	Coumestrol	-8.1
9	Thaspine	-8
10	Parthenicin	-7.9
11	Illudine M	-7.9
12	ATP (Co-crystal)	-7.9
13	Triptolide	-7.6

**Table 2:** Physicochemical and drug likeness of screened compounds.

Compound Name	Mol. wt	cLogP	cLogS	H-Accep tors	H-Donors	Drug likeness	Muta genic	Tumori genic	Rep. Effective	Irritant	Drug Score	Total Surface Area	Polar Surface Area
Toyocamycin	291.266	-1.4642	-3.412	9	4	-5.4705	N	N	N	N	0.265084	204.12	150.44
Nanaomycin	302.281	1.0284	-3.032	6	2	2.1075	N	N	N	N	0.842116	209.91	100.9
Corynanthin	354.448	2.3512	-3.065	5	2	1.5035	N	N	N	N	0.762417	258.65	65.56
Ehnahydrochloride	277.371	2.2338	-3.073	6	2	-13.836	N	N	N	N	0.439682	226.53	89.85
Medicarpin	270.283	3.1657	-3.031	4	1	-0.8225	N	N	H	N	0.332054	193.39	47.92
Pentoxifyllin	278.311	0.9925	-2.176	7	0	-1.5832	H	N	H	H	0.11778	213.39	75.51
Taxifolin	304.253	0.9579	-1.945	7	5	0.44477	N	N	N	N	0.745825	204.02	127.45
Coumestrol	268.224	2.8407	-4.345	5	2	-0.4041	H	N	H	N	0.192383	184.26	79.9
Thaspine	369.372	2.5732	-4.132	7	0	2.7556	N	N	N	N	0.724717	268.38	74.3
Parthenicin	262.304	0.9307	-2.457	4	1	-5.759	N	N	N	H	0.279694	184.46	63.6
Illudine M	248.321	1.6644	-2.145	3	2	1.4572	N	N	N	N	0.845633	171.37	57.53
N = No; H = High
